# The Potential of the Nose-to-Brain Delivery of PACAP for the Treatment of Neuronal Disease

**DOI:** 10.3390/pharmaceutics15082032

**Published:** 2023-07-28

**Authors:** Asma Cherait, William A. Banks, David Vaudry

**Affiliations:** 1 Univ Rouen Normandie, Inserm U1245, Medical Faculty, Normandie Univ, F-76000 Rouen, France; a.cherait@esa-mosta.dz; 2 Department of Second Cycle, Higher School of Agronomy Mostaganem, Mostaganem 27000, Algeria; 3 Laboratory of Cellular Toxicology, Department of Biology, Faculty of Sciences, University of Badji Mokhtar Annaba, B.P. 12, Annaba 23000, Algeria; 4 Geriatric Research Educational and Clinical Center, Veterans Affairs Puget Sound Health Care System, Seattle, WA 98108, USA; 5 Division of Gerontology and Geriatric Medicine, Department of Medicine, University of Washington School of Medicine, Seattle, WA 98104, USA; 6 Univ Rouen Normandie, Inserm US51, Regional Cell Imaging Platform of Normandy (PRIMACEN), Sciences and Technologies Faculty, Normandie Univ, F-76000 Rouen, France

**Keywords:** pituitary adenylate cyclase-activating polypeptide, intranasal delivery route, neuronal diseases, neuroprotection, apoptosis, inflammation, redox state, cerebral plasticity

## Abstract

Research on the neuroprotective effect of pituitary adenylate cyclase-activating polypeptide (PACAP) and its use as a therapeutic agent has grown over the past 30 years. Both in vitro and in vivo experiments have shown that PACAP exerts a strong neuroprotective effect in many central and peripheral neuronal diseases. Various delivery routes have been employed from intravenous (IV) injections to intracerebroventricular (ICV) administration, leading either to systemic or topical delivery of the peptide. Over the last decade, a growing interest in the use of intranasal (IN) administration of PACAP and other therapeutic agents has emerged as an alternative delivery route to target the brain. The aim of this review is to summarize the findings on the neuroprotective effect of PACAP and to discuss how the IN administration of PACAP could contribute to target the effects of this pleiotropic peptide.

## 1. Introduction

Pituitary adenylate cyclase-activating polypeptide (PACAP) was originally isolated from ovine hypothalamic extracts for its ability to stimulate cAMP formation in rat anterior pituitary cells [1]. PACAP belongs to the secretin/glucagon/vasoactive intestinal peptide superfamily, which includes vasoactive intestinal peptide (VIP), glucagon, glucagon-like peptide-1 and -2 (GLP1, GLP2), glucose-dependent insulinotropic polypeptide (GIP), growth hormone-releasing hormone (GHRH) and peptide histidine isoleucine (PHI) [2,3]. PACAP presents two forms, i.e., a 38 amino acid polypeptide, referred to as PACAP in this review, which can be cleaved by prohormone convertases, to generate a 27 amino acid peptide [4]. However, in the brain, PACAP38 represents approximatively 90% of total PACAP [5].

PACAP acts via three G-protein-coupled receptors widely distributed in the organism, i.e., the PACAP-selective PAC1 receptor, and the VIP/PACAP mutual VPAC1 and VPAC2 receptors [6,7]. The various PACAP biological effects, depend on the ligand concentration, the tissue cell type and the stage of development, alongside with the expression of various receptor isoforms responsible for the activation of several signal transduction pathways such as adenylyl cyclase, phospholipase C, protein kinase A, PI3K/Akt and mitogen-activated protein kinase, and their outcomes, leading sometimes to opposite effects [6,8,9,10].

If PACAP was initially discovered as an hypophysiotropic neurohormone, it is now widely recognized to regulate the metabolism, respiratory, reproductive, cardiovascular, immune functions, etc., in health and disease, by controlling important biological processes such as cell proliferation, differentiation, migration and apoptosis [2,9,11]. On peripheral and central nervous systems, PACAP acts as a neurohormone, neurotransmitter and neurotrophic factor [2]. Furthermore, numerous studies have also highlighted the remarkable neuroprotective effects of PACAP [12,13,14]. This review provides a brief overview of these neuroprotective actions of PACAP in several neuronal diseases. More specifically, we focus on the ability of PACAP to counteract some deleterious mechanisms involved in brain injury such as oxidative stress, inflammation and apoptosis. We then examine the potential of intranasal administration of PACAP as a delivery route for the treatment of these neuronal diseases and the elements which can impair its clinical use are discussed.

## 2. Neuroprotective Effects of PACAP for Treatment of Brain Insults

Over the past 30 years, PACAP has been shown to exert strong neurotrophic and neuroprotective effects in both in vitro and in vivo models of various neuropathologies [15,16,17] alongside with its CNS antimicrobial activity [18].

**Figure 1:**
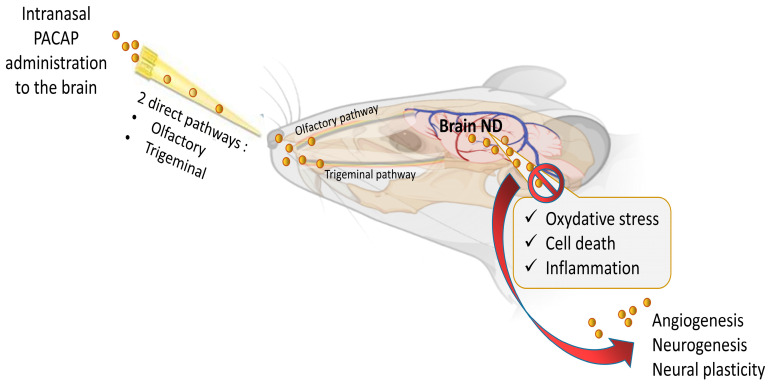
Multi-neuroprotective mechanisms of PACAP administrated intranasally in neurodegenerative disease (ND). The figure was created with BioRender.com and Microsoft PowerPoint 2021.

This neuropeptide plays an important role, and its deficits contribute to various neurodegenerative and neurological diseases (ND). For example, in Alzheimer’s disease (AD), PACAP levels in cerebrospinal fluid (CSF) samples and brain tissue of human patients are low and correlated with a variety of cognitive measures in mild cognitive impairment and dementia stages [19]. In rodent models, PACAP treatment slows AD progression, protecting neurons from the toxicity of β-amyloid 42 oligomers through a boost of the expression of sirtuin 3, which in turn enhances mitochondrial function [12,20]. Furthermore, this neuroprotective action of PACAP seems at least in part to occur via its interaction with β-amyloid [21]. The neuroprotection of PACAP has also been demonstrated in a rat model of Parkinson’s disease, where administration of the peptide prevents nigral dopaminergic neuronal degeneration, slows down cognitive decline and rescues behavioral deficits via an increase in the levels of dopamine and of Parkinson’s disease protein 7 (PARK7) [22,23]. In rodent models of hemorrhagic and ischemic stroke (subarachnoid hemorrhage, tMCAO, pMCAO), PACAP decreases neuronal loss and promotes functional recovery [14,24,25,26]. These strong protective effects come from the capacity of the peptide to reduce in a complementary way glutamatergic excitoxicity, oxidative stress, apoptosis and inflammation (Figure 1) [14,27,28,29]. Finally, it can be mentioned that PACAP improves cognitive impairment in vascular dementia through its ability to regulate synaptic plasticity and to inhibit apoptosis [30].

In some cases, as reported for amyotrophic lateral sclerosis (ALS) and multiple sclerosis (MS) [31], the neurodegenerative disease is amplified by the deficiency of endogenous PACAP, suggesting that an exogenous administration of the peptide may be useful for the treatment of the pathology.

Based on these data, it appears clearly that PACAP plays an essential neuroprotective role in response to various brain insults, such as cerebral ischemia, subarachnoid hemorrhage and traumatic brain injury, as well as in several neurodegenerative diseases, such as Parkinson’s disease, Alzheimer’s disease, Huntington’s disease and amyotrophic lateral sclerosis [14,23,26,32,33,34]. Interestingly, it has been shown that in these diseases, PACAP administration counteracts various pathological processes such as oxidative stress, neuronal cell death and inflammatory responses as shown in Figure 1.

## 3. Mechanisms Involved in the Neuroprotective Effects of PACAP

PACAP influences various major common pathological hallmarks of neuronal diseases, including unbalanced redox state, cell survivor and death, and inflammation. For instance, several studies have demonstrated its strong antioxidative effect. Indeed, PACAP can stimulate the expression of antioxidant detoxifying machinery through its actions on peroxiredoxin 2 and 5, thioredoxin reductase, glutathione, catalase and superoxide dismutase [35,36,37,38,39], and to reduce the production of pro-oxidant factors through its actions on nitric oxide (NO) synthase (NOS 1 and 2), NADPH oxidase, and lactate dehydrogenase [14,28,40,41].

PACAP has the capacity to reduce the three forms of neuronal cell death: apoptosis, necrosis and autophagy.

The effect of PACAP on apoptosis was the first form of cell death investigated [11,42]. Ever since, various in vitro and in vivo studies have shown that PACAP prevents neuronal apoptosis through the regulation of the Bcl family members via the activation of the PAC1 receptor and several downstream complementary transduction pathways such as the PKA pathway [26], the MAPK pathway [43,44,45] and the CREB-Bcl-2 pathway [46]. This results in the inhibition of proapoptotic factors such as Bax, Bad, caspase-9 and caspase-3, promoting anti-apoptotic events such as the increase in Bcl-2 expression [20,27,47].

Regarding the autophagic process, PACAP decreases the autophagic activity in Parkinson’s disease through the production of the LC3-II complex, the increase in p62 levels and the reduction formation of autophagic vacuoles [48]. PACAP also reduces hypoxia-induced autophagic cell death in an in vitro model of amyotrophic lateral sclerosis, by activating the MAPK/ERK signalling cascade [49].

Necrotic cell death includes both uncontrolled cell death (necrosis) and controlled cell death (necroptosis, pyroptosis, ferroptosis and parthonatos), but only the uncontrolled type will be addressed in this paper. Necrotic cell death is typically characterized by energy failure, ROS production, loss of membrane permeability, swelling, and membrane rupture. It is also associated with a strong inflammatory response, which is common in neuronal diseases and associated with subsequent pathology [50,51]. PACAP can counteract necroinflammation and promote neuronal survival by its immunoregulatory properties [28]. PACAP also prevents cell swelling and membrane rupture through inhibition of the expression of aquaporin 4 and SUR1 [14,52].

PACAP controls the inflammatory process by decreasing various proinflammatory factors, including tumor necrosis factor-α (TNF-α), macrophage inflammatory protein-1 alpha (MIP-1α), some interleukins such as IL-6, IL-8 and IL-12, the receptor for advanced glycation end-products (RAGE), and the transcription factor NF-κB [14,15,20,27,29,53]. Altogether, these effects of PACAP tend to cause the redirection of the microglial response toward a neuroprotective M2 phenotype.

Besides its neuroprotective effects, PACAP promotes brain repair through stimulation of neurogenesis, synaptic plasticity and angiogenesis [13,54,55]. This regenerative effect is essentially due to the ability of PACAP to stimulate the expression of genes such as brain-derived neurotrophic factor (BDNF), solute carrier family 16 member 7 (Slc16a7), neuronal differentiation 1 (NEUROD1), vascular endothelial growth factor A (VEGFA), homer scaffold protein 1 (HOMER 1), diazepam-binding inhibitor (DBI/ACBP), and sirtuin 3, as well as others [14]. Probably linked to the release of these trophic factors, PACAP increases the axonal outgrowth plasticity, modulates synaptic transmission [56,57], and promotes dendritic spine maturation and morphogenesis [58]. Concordantly, PACAP rescues hippocampal synaptic plasticity through stimulation of adenylate cyclase, and corrects abnormal metabotropic glutamate receptor-mediated long-term depression in the hippocampal neurons of Fragile X Mental Retardation 1 (Fmr1) knockout mice, a Fragile X Syndrome model [59]. Furthermore, PACAP promotes proliferation of neural stem cells isolated from the lateral ventricle wall of the adult mouse brain via the protein kinase C pathway [60]. Taken together, all these studies strongly suggest that PACAP administration has a therapeutic potential for the treatment of neurological diseases.

## 4. The Challenges in PACAP Delivery to the Brain

**Figure 2:**
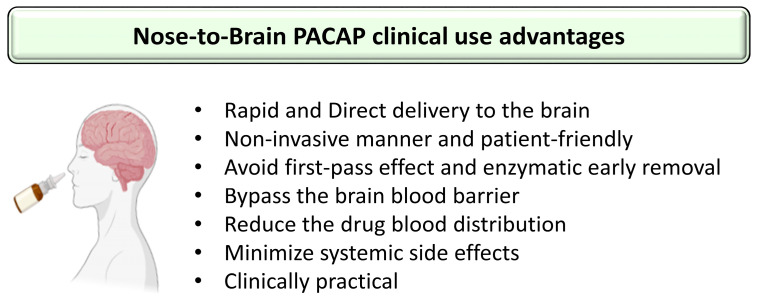
Summary of the advantages using nose-to-brain PACAP administration in the clinic. The figure was created using BioRender.com and Microsoft PowerPoint 2021.

The treatment of neuronal diseases represents a challenge because of the blood–brain barrier (BBB) which limits the ability of most biomolecules to reach the central nervous system (CNS) in therapeutic amounts. Essentially, the BBB is a complex and selective interface regulating the ability of molecules to cross from blood to brain and vice versa [61,62]. Regarding PACAP38, it has saturable components to both its blood-to-brain and brain-to-blood transport (PTS-6) [63], whereas PACAP27 crosses in the blood-to-brain direction by transmembrane diffusion, but has a saturable component to its brain-to-blood transport [64,65]. Various systemic or topical administration routes of PACAP have been reported such as ICV, IP and IV [7,28,66]. PACAP directly injected into the brain can not be considered a readily available translational option because of its extremely invasive nature and its clinically impractical application [67]. PACAP administrated IP and IV can circulate through the blood to reach the BBB. However, in the blood, PACAP has a very short half-life of less than 5 min because of its rapid degradation by the dipeptidylpeptidase IV (DPP IV), an exopeptidase that cleaves X-proline or X-alanine dipeptides from the N-terminals of polypeptides [68,69]. This means that PACAP’s main problem for use as a therapeutic agent is not its capacity to cross the BBB but its rapid degradation in blood and thereby its low bioavailability. Furthermore, PACAP receptors are widely expressed in peripheral tissues where the peptide has a plethora of functions, raising the potential for undesirable peripheral side effects. For example, an IV injection of PACAP may induce anorexia, increase body temperature, dysregulate the cardiovascular system or promote insulin levels, etc. [70,71,72,73]. Therefore, using a nose-to-brain delivery route (NtB), which is also referred to as intranasal administration to the brain (IN) in this manuscript, allows the peptide to reach the brain without passing through the blood circulation and represents a huge asset in neuronal diseases treatment efficiency. The mode of IN administration leads to a direct delivery of the molecules of interest from the nasal cavity to the brain significantly through the olfactory and trigeminal pathways (Figure 1), avoiding systemic exposure to the peptide [74]. In addition, the peptide drug concentrations of this delivery route can be similar or higher to the profile of systemic administration [67,75,76] with some 100-fold concentration increases in multiple brain regions [77]. The other benefits of PACAP administration, as shown in Figure 2, are its potential rapid delivery to the brain within 5 to 15 min and the non-invasive aspect of this clinically applicable administration route [7,20].

## 5. The Mechanism of the PACAP Nose-to-Brain Route

The nasal cavity is divided into three distinct regions: (1) the vestibule region, the anterior part of the nasal cavity, lined with cilia on the surface of epithelial cells; (2) the respiratory region, the largest area of the nasal cavity, lined by a ciliated epithelium, interspersed with mucus-secreting goblet cells; and (3) the olfactory region, the upper region of nasal cavity lined by ciliated olfactory cells [78]. Before reaching the brain, PACAP follows multiple pathways through these different nasal cavity regions and could be the subject of early elimination under the action of nasal enzymes [79]. On its way, PACAP has to deal firstly, in the vestibular region, with the mucociliary clearance of the epithelial cilia cells. The coordinated movement of this hair-like cilia structure play an important role in draining and cleaning the mucus and could impede PACAP NtB delivery, favoring its removal into the gastrointestinal tract by way of the nasopharynx. In the respiratory region, a small amount of the peptide can reach the brain either indirectly after entering the circulation and crossing the BBB or directly via the trigeminal axonal transport which extends to the brain stem after crossing the lateral respiratory epithelium [78]. However, the direct NtB distribution of PACAP occurs mostly through the olfactory and trigeminal pathways across the cribriform plate into the olfactory region. These neurons have receptors that allow PACAP transport into the cerebrospinal fluid and olfactory bulb and then its distribution to other brain regions through various neural connections [80]. It is this route through the olfactory nerve that may be utilized for optimal delivery of PACAP to the central nervous system. It is important to note that the rapidity of peptide brain delivery via the olfactory and trigeminal pathways depends on the nature of the transport solicited, taking either minutes via the perineural paracellular transport from the sub-mucosal space to the CSF compartment or hours through the intracellular axonal transport [81]. Possible mechanisms of transport may also involve direct drug delivery to the brain through the lymphatic system and the vasculature adjacent to the CSF [80]. Nevertheless, the exact PACAP “highway” to the brain remains undetermined.

## 6. Preclinical Studies Highlighting the Efficiency of the Nose-to-Brain (NtB) Route to Deliver PACAP

After PACAP NtB administration, the highest amount of PACAP uptake is observed in the occipital cortex and striatum regions with approximately 2 to 4% of the administered dose per gram of brain [82]. A significant amount of the exogenous PACAP is also found in other brain regions such as the hippocampus and hypothalamus. Additionally, what is very important is that the quantity of PACAP reaching the brain after NtB administration seems sufficient to improve cognitive and functional performance in various models of neuronal degeneration [14,82].

Indeed, in the APP[V717I] Alzheimer’s disease mouse model, NtB PACAP administration improves cognitive performances and increases the processing of APP through the non-amyloidogenic pathway. PACAP activates α-secretase, which results in an increased secretion of neuroprotective sAPP-α and a decreased secretion of sAPP-β [20]. PACAP also stimulates brain-derived neurotrophic factor (BDNF) mRNA and protein levels by inducing CREB phosphorylation, and reduces inflammation via a decrease in RAGE expression, which in turn inhibits Aβ transport into the brain. Additionally, PACAP daily delivery leads to an increased expression of its own gene [83] and of its specific PAC1 receptor [20], which ultimately should potentiate the neuroprotective effect of the exogenous administered peptide.

In the R6/1 mice Huntington’s disease model, PACAP daily NtB delivery enhances cognitive performances. The administration of PACAP results in a reduction in huntingtin mutant aggregate formation and an increase in vesicular glutamate transporter 1, postsynaptic density protein 95 (PSD-95) and BDNF expression in the hippocampus. These effects occur via the activation of the PAC1 receptor whose expression is restored after PACAP treatment [84].

In a bilateral common carotid stenosis (BCAS) mouse model of vascular dementia, PACAP NtB activates the PAC1 receptor, which increases the expression of BDNF, PSD-95 and Sirt3, leading to a protective effect on synaptic integrity and improved plasticity [30]. The same authors have also shown, with the immortalized mouse hippocampal neuronal cell line HT22, that PACAP increases the expression of the apoptosis inhibitor Bcl-2 and of the deacetylase sirtuin family member Sirt3, which protects mitochondrial homeostasis and favors cell survival.

In a transgenic mouse model of spinobulbar muscular atrophy (SBMA), a motor neuron disease caused by misfolded protein aggregation, NtB administration of a PACAP analog reduces Ser96 phosphorylation of the polyglutamine (polyQ) expansion of the androgen receptor. This decreases protein stability and toxicity, leading to a better outcome [85].

These few examples and the other studies reported in Table 1, illustrate the growing interest for the NtB delivery of PACAP in the treatment of neuronal diseases (Table 1) since 2011. However, this route of administration has also shown promising results with other molecules [4,69,86,87] and it is even now under clinical trials for some of them, such as insulin (NCT01767909, NCT05006599, etc.) [88,89] or Protollin [90], as part of a protocol for the treatment of Alzheimer’s disease. The NtB delivery route has also shown its efficiency and safety in the treatment of children with cerebral palsy, using neural stem cells (NCT03005249) [91].

## 7. What Could Impair the Use of the PACAP Nose-to-Brain (NtB) Delivery Route in the Clinic?

**Figure 3:**
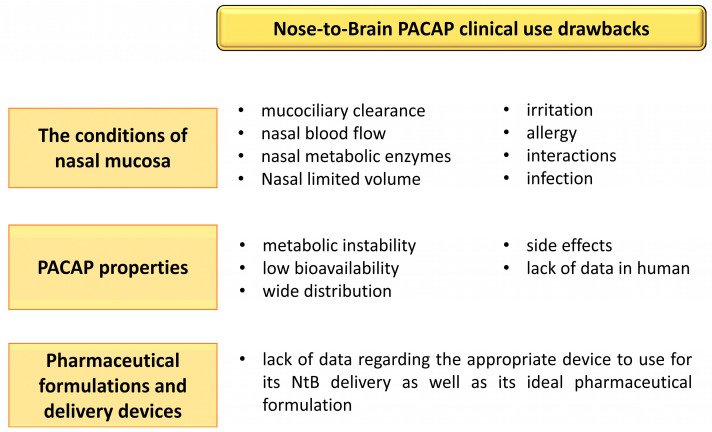
Summary of the difficulties associated with the use of nose-to-brain (NtB) PACAP administration in clinic. The figure was created using Microsoft PowerPoint 2021.

As with any delivery route of a therapeutic compound, the intranasal route to the brain has advantages but unfortunately also drawbacks. Regarding PACAP NtB delivery, limitations can be divided into three categories (Figure 3): the conditions of the nasal mucosa, the PACAP proprieties, and the pharmaceutical formulations and delivery devices.

### 7.1. Influence of the Nasal Mucosa Condition in PACAP Absorption

PACAP absorption through the nasal mucosa is influenced by various factors such as (1) the mucociliary clearance [92], which could transfer PACAP to the nasopharynx and eventually, to the gastrointestinal tract; (2) the nasal blood flow which could decrease PACAP absorption or even promote its systemic distribution [77,93]; (3) the behavior of the molecule in its environment, whose nature of interactions is unpredictable; and (4) the nasal metabolism and enzymatic degradation of the peptide [94], because even if the NtB administration avoids the first-pass effect, the presence of metabolic enzymes (proteases, nucleases, etc.) in nasal tissues can cause its degradation. An allergy, an infection, an irritation, or the use of other nasally delivered medications may also influence PACAP absorption and efficiency, as is the case of any drug delivered by this route of administration. However, these limitations can be overcome via appropriate nasal pharmaceutical formulations of PACAP, which will probably play an important role in its safety, ease of use and comfort. Indeed, various studies have shown the importance of an optimized formulation for drug nasal delivery so as to avoid nasal irritation and ensure good tolerance of treated subjects [93,95]. The suitable administration technique and proper device that deposits the peptide in the posterior and upper region of nasal cavity will favor its brain uptake and immediate action, thanks to the direct anatomic pathway between the brain and the nasal nerves of the neuroepithelium. Targeting this region of the olfactory system also avoids mucociliary clearance of PACAP and its subsequent migration to systemic circulation [80,96]. Additionally, this surface area is deeply vascularized with a porous endothelial membrane that favors brain delivery [80].

### 7.2. PACAP Properties

**Figure 4:**
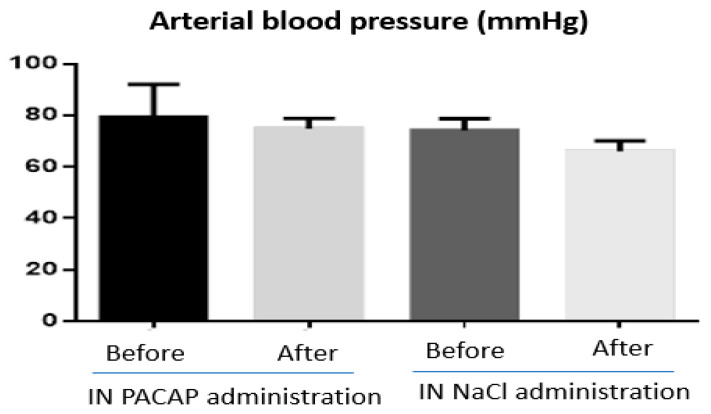
Arterial blood pressure measurement (mmHg) before and after nose-to-brain (NtB) delivery of PACAP (n = 5) and NaCl (n = 4) in mice. All animals were treated with a single NtB administration of 10 µL of PACAP (1 µg/µL) or Nacl (0.9%). The figure was created using GraphPad Prism 6. Statistical analysis of the results revealed no significant differences.

The physicochemical characteristics and biological activities of PACAP represent a real challenge for its medicinal use. In particular its metabolic instability in blood, low bioavailability, wide distribution, numerous side effects and lack of data in humans impairs the development of clinical applications. However, as mentioned above, preclinical studies have demonstrated that despite the metabolic instability of PACAP, the amount of peptide that reaches the brain after NtB administration is sufficient to exert a strong neuroprotective effect [14,20,82] with fewer side effects compared to systemic administration. Indeed, in our recent study, NtB PACAP administration had no influence on body weight and food intake [14] or on blood pressure (Figure 4; unpublished work) in mice. Furthermore, Doberer et al. [97] have reported that inhaled PACAP, with a possible NtB and nose-to-blood absorption, was well tolerated in human subjects without systemic side-effects (blood pressure, pulse rate or skin blood flux) or headache. This is consistent with a clinical study that provided evidence that the NtB delivery route of drugs is safer with fewer side effects than other administration methods such as oral and rectal [95]. However, until now, there is no clinical trial for the use of PACAP NtB delivery for the treatment of neurodegenerative diseases. This could be explained mostly by the potential local side effects of PACAP and the lack of evidence regarding its human efficiency and innocuity. We know, however, that the receptors for PACAP are expressed in the human brain [98] and that in macaque brain, PACAP can inhibit apoptosis [99,100,101], which should encourage clinical studies.

To further reduce the risk of potential side effects of PACAP NtB administration and to amplify its neuroprotective response, it would now be helpful to target only the brain areas responsible for the neuronal diseases to be treated without affecting the other brain structures. For this purpose, the use of excipients such as cyclodextrins (CD) can influence greatly the brain regions that take up PACAP [82]. The application of a nanosized delivery system or the use of AAV has also been proposed for targeted PACAP brain delivery [102,103,104,105,106] as highlighted in the following undersection. In combination with this, some metabolically stable PACAP analogues with improved pharmacokinetic properties, good tolerability and high selectivity for one of the PACAP receptors [69,107,108,109] could contribute to the development of the ideal PACAP nasal formulation and its delivery device.

### 7.3. Pharmaceutical Formulations and Delivery Devices

An optimized galenic development for PACAP NtB delivery might thus diminish PACAP side effects, enhance its absorption rate and increase its efficiency. As it is well known, the density, the velocity and the pH of formulations can impact the absorption of a peptide, cause mucosa irritation and favor pathogenic bacteria growth [110,111]. For instance, a pH between 4.5 and 6.5 is optimum to avoid nasal irritation [112].

The use of excipients, including absorption enhancers, mucoadhesives, enzyme inhibitors, liposomes or cell penetrating peptides should increase PACAP NtB absorption [94] since the use of cyclodextrins, an absorption enhancer, improves PACAP nasal incorporation and brain targeting. Indeed, NtB of 4 µL of lactated Ringer’s solution containing 1% bovine serum albumin and 500,000 cpm/μL of radioactively labeled iodinated PACAP (I-PACAP) in presence or absence of 5% β-cyclodextrin, (2-Hydroxypropyl)-β-cyclodextrin or α-Cyclodextrin was administrated to aged SAMP8 mice, an animal model of Alzheimer’s disease. To perform that, a small cannula attached to a 10 μL syringe was pushed to the depth of the cribriform plate through the two nares. The result obtained showed a distinct increase in I-PACAP brain distribution with an improvement on memory performance compared to the peptide administered alone [82]. Furthermore, the I-PACAP shows a preferential distribution in some brain regions based on the type of CD used and the excipient protects the peptide from enzymatic degradation [82,94]. The use of β-cyclodextrins can enhance greatly the uptake of PACAP in the brain occipital cortex and hypothalamus, whereas the use of α-cyclodextrin promotes its distribution into the olfactory bulb and decreased its uptake into the occipital cortex and striatum. The use of (2-hydropropyl)-β-cyclodextrin increased its absorption by the thalamus and decreased its uptake by the striatum [82]. In addition to cyclodextrin, mucoadhesive excipients such as viscous formulations, mucoadhesive polymers, hydrogels or in situ gelations could be useful to increase the contact duration with nasal mucosa, both enhancing bioavailability of the molecule and reducing mucociliary clearance [112]. The use of epinephrine as a local vasoconstrictor can also help to decrease nose-to-blood absorption, reduce the systemic side effects and enhance the brain exposure via olfactory and trigeminal pathways [113].

The nanocarrier-based systems have shown their efficiency in facilitating peptide brain-specific delivery with an excellent characteristic of biocompatibility and biodegradability and peptide controlled release [114,115,116]. Nanocarriers could also be an effective and non-invasive method for PACAP NtB delivery, preventing metabolic degradation via peptide encapsulation. These PACAP transporter tools can be designed with specific properties, through the attachment of various functionalizing agents, to target a distinct brain region, enhancing efficiency of PACAP delivery and reducing potential undesirable effects [114,117]. For example, we can cite a liposome functionalized with a cell-penetrating peptide, the membrane-perturbing domain in glycoprotein H (gH) of Herpes simplex virus 1. This modification has generated a gH625 liposome that is able to promote PACAP brain uptake in a non-toxic manner, both in vitro in a rat BBB model and in vivo in mice [117]. Another example is the use of a nanosized polymer that can protect against enzymatic degradation of the peptide, increasing NtB permeation and having controlled release [116,118,119]. Chitosan, a commonly used natural cationic polysaccharide, has bioadhesive properties and can open tight junctions, thus increasing drug permeability by the NtB route and reducing mucociliary clearance [105]. These properties can be enhanced with transferrin-decorated chitosan nanoparticles [120]. Combining a surface-modified chitosan with transferrin, which uses receptor-mediated endocytosis to cross membranes, increases both the rapidity of passage through the epithelial cell layer and drug cellular uptake [120]. As a final example, synthetic poly (ethylene glycol)-poly (lactic acid) (PEG-PLA) nanoparticles can be coupled with wheat germ agglutinin, an olfactory targeting functionalizing agent, to mediate VIP NtB transport, resulting in a better neuroprotection than the peptide when administrated alone [121]; this strategy should be applicable to PACAP as well as other peptides. Various other ligands that have been used to coat nanocarriers to enhance brain deliver include glutathione, biotin, HIV-1 TAT protein, lactoferrin and albumin [118,122,123].

The use of viral delivery vectors could also be a possible option. PACAP encoding adeno-associated virus enhanced the survival of rat primary cortical neurons against neurotoxic injury in comparison to either lipofection-mediated PACAP delivery using DOTAP liposomal transfection reagent or untransfected cells [124]. The use of AAV technology has several advantages such as its CNS tropism, transduction efficiency, stability and biosafety. NtB delivery offers several advantages compared to traditional routes [106], as systemic delivery of AA or its direct brain injection is associated with toxicities (immunotoxicity, neurotoxicity, etc.) and peripheral “off-target effects” [125,126,127], especially when higher doses of the helper virus is given [126].

The NtB route of administration could assure an optimum AAV-mediated PACAP delivery that continuously releases the peptide from a localized brain area. The AAV serotype 9 variants AAV9 MaCPNS1/2 could be a serious candidate for that, achieving a high transduction in astrocytes and neurons in various brain regions [128]. In theory, these could be engineered to target specific regions of the CNS. Different serotypes transduce neurons at different degrees, such as AAV1, AAV2, AAV5, AAV8, and AAV rhesus isolate10 (AAVrh.10) [127,129]. However, further work needs to be done before clinical applications can be considered, including determining dosing parameters, which AAV serotypes have the potential for distal transduction and thus peripheral effects, the impacts on immune responses and the effects of chronic PACAP administration.

For effective treatment of ND, it may be necessary to consider using PACAP in combination with other neuroprotective agents. For this purpose, the use of self-assembled cubosome nanoparticles presents an innovative strategy that has already been tested by linking the neuroprotectant docosahexaenoic acid (DHA) with PACAP to create a new bioactive amphiphile PACAP-DHA [102].

The nature of the pharmaceutical formulation device that will be used for PACAP NtB delivery, such as nasal drops, nasal sprays, aerosol sprays or insufflators, may also affect peptide efficiency. For example, the use of nasal drops can result in a rapid nasal drainage with a potential incorrect dosage [130], whereas the use of powder sprays can cause nasal irritation [130,131]. The same is true for excipients such as antioxidants, preservatives and flavorings [93,132]. As reviewed by Triveno et al. [92], various devices for intranasal delivery to the brain have already been tested in clinical trials for a range of drugs, including peptides. Among these, we can cite the Precision Olfactory Delivery (POD^®^), SipNose^TM^ and Optimist^TM^ technologies which target the olfactory epithelium, and, at least for the last device, minimize the risk of lung deposition; the Aero Pump^TM^ system which limits the risk of contamination; or the ViaNase^TM^ apparatus which allows precise electronic dosing, targets the delivery to the olfactory epithelium and maximizes NtB transport. All these technologies have shown the incredible potential to influence the delivery of molecules to the brain and their efficiency. Some other promising inventions are still in preclinical testing, such as the Aeroneb^®^ Pro, the Naltos^TM^, the Versidoser^®^ and the VRX2^TM^ [92]. So far, PACAP has mainly been administered in the form of small drops, for example, a volume of 5 μL/nostril of 1 μg of PACAP dissolved in 1 µL of water solution containing 7.5 µg of NaCl, 1.7 µg of citric acid monohydrate, 3 µg of disodium phosphate dehydrate, and 0.2 µg of benzalkonium chloride solution (50%) [20], or using a volume of 10 µL/mouse of 1 µg/µL, 1 ng/µL, 1 pg/µL and 1 fg/µL of PACAP solution dissolved in 0.9% NaCl [14]. The use of such innovative devices could help to minimize PACAP potential adverse effects and enhance its brain delivery and neuroprotective efficiency. Unfortunately, these systems often have no equivalent for preclinical research in rodents that would allow assessment of their effectiveness for NtB delivery.

All these developments open fascinating and realistic perspectives, but the main limitations for the use of NtB PACAP delivery remains the lack of data on efficacy and safety in humans, the absence of data on the various devices, and the development of an ideal pharmaceutical formulation. These issues should now be the research priorities for future clinical use. Clinical studies should be possible based on all the preclinical data accumulated over the past 30 years together with the use of predictive modelling to design safe PACAP NtB delivery systems.

## 8. Conclusions

To conclude this review, the use of the NtB route for delivery to the brain of various compounds has increased dramatically over the last few years. Preclinical studies using PACAP NtB delivery have shown that it is an efficient route of administration for the treatment of neuronal diseases in various rodent models. The results indicate that NtB is a valuable alternative route to the more traditional ones, bringing PACAP to the CNS non-invasively, circumventing the rapid degradation in blood, and delivering an amount of peptide to the brain sufficient for a beneficial outcome in various neuronal diseases. NtB PACAP delivery also limits peripheral side effects because of the limited quantity of peptide that enters the systemic circulation [4]. However, the PACAP NtB delivery route presents various drawbacks which must still be overcome for a successful translational application to the clinic; to achieve that, several pharmacological and toxicological studies are required. Finally, it is now time that clinicians paid attention to this peptide to start clinical protocols in order to really assess the therapeutic potential of the NtB PACAP delivery route for the treatment of neuronal diseases in humans.

## Figures and Tables

**Table 1:** Examples of preclinical studies using the intranasal administration to the brain of PACAP for the putative treatment of neuronal diseases. This list was established after a PubMed literature search to identify studies related to PACAP nose-to-brain/intranasal administration in neuronal disease.

Neuronal Disease	Animal Model Disease	Reference	Outcomes
Stroke	PMCAO and tMCAO mouse model	[14]	Infarct volume reduction and functional recovery.
Alzheimer	Transgenic APP (amyloid precursor protein) mouse model	[20]	Cognitive function improvement and stimulation of non-amyloidogenic pathway of APP[V717I]; Enhanced Aβ-degrading enzyme neprilysin, BDNF and Bcl-2 protein expression; reduced amyloid β-peptide (Aβ) transporter receptor expression.
	SAMP8 mice	[82]	Highest PACAP uptake by occipital cortex and striatum in comparison to other brain regions with enough therapeutic amounts of PACAP to enhance memory performance. Addition of cyclodextrins may contribute to targeting specific brain regions with PACAP.
Huntington	R6/1 mice and HdhQ7/Q111 mouse model	[84]	Enhancement of plasticity and cognitive performances via an increase in VGlut-1, PSD95, BDNF and PAC1 levels; reduction in the formation of mutant huntingtin aggregates.
Vascular dementia	Bilateral common carotid stenosis knock-in SBMA mouse model	[30]	Improvement of synaptic plasticity and of cell survival by increasing expression of BDNF, PSD-95, Sirt3 and Bcl2.
Spinal bulbar muscular atrophy	Knock-in SBMA mice	[85]	Improvement of the disease outcome. Reduction in Ser^96^ phosphorylation of polyQ androgen receptor, which promotes its degradation.

## Data Availability

Data are available from the corresponding author on request.
